# ChIP-Atlas 3.0: a data-mining suite to explore chromosome architecture together with large-scale regulome data

**DOI:** 10.1093/nar/gkae358

**Published:** 2024-05-16

**Authors:** Zhaonan Zou, Tazro Ohta, Shinya Oki

**Affiliations:** Institute of Resource Development and Analysis, Kumamoto University, 2-2-1 Honjo, Chuo-ku, Kumamoto 860-0811, Japan; Department of Drug Discovery Medicine, Kyoto University Graduate School of Medicine, 53 Shogoin Kawahara-cho, Sakyo-ku, Kyoto 606-8507, Japan; Institute for Advanced Academic Research, Chiba University,1-33 Yayoi-cho, Inage-ku, Chiba 263-8522, Japan; Department of Artificial Intelligence Medicine, Graduate School of Medicine, Chiba University, 1-33 Yayoi-cho, Inage-ku, Chiba 263-8522, Japan; Database Center for Life Science, Joint Support-Center for Data Science Research, Research Organization of Information and Systems, Yata 1111, Mishima, Shizuoka 411-8540, Japan; Institute of Resource Development and Analysis, Kumamoto University, 2-2-1 Honjo, Chuo-ku, Kumamoto 860-0811, Japan; Department of Drug Discovery Medicine, Kyoto University Graduate School of Medicine, 53 Shogoin Kawahara-cho, Sakyo-ku, Kyoto 606-8507, Japan

## Abstract

ChIP-Atlas (https://chip-atlas.org/) presents a suite of data-mining tools for analyzing epigenomic landscapes, powered by the comprehensive integration of over 376 000 public ChIP-seq, ATAC-seq, DNase-seq and Bisulfite-seq experiments from six representative model organisms. To unravel the intricacies of chromatin architecture that mediates the regulome-initiated generation of transcriptional and phenotypic diversity within cells, we report ChIP-Atlas 3.0 that enhances clarity by incorporating additional tracks for genomic and epigenomic features within a newly consolidated ‘annotation track’ section. The tracks include chromosomal conformation (Hi-C and eQTL datasets), transcriptional regulatory elements (ChromHMM and FANTOM5 enhancers), and genomic variants associated with diseases and phenotypes (GWAS SNPs and ClinVar variants). These annotation tracks are easily accessible alongside other experimental tracks, facilitating better elucidation of chromatin architecture underlying the diversification of transcriptional and phenotypic traits. Furthermore, ‘Diff Analysis,’ a new online tool, compares the query epigenome data to identify differentially bound, accessible, and methylated regions using ChIP-seq, ATAC-seq and DNase-seq, and Bisulfite-seq datasets, respectively. The integration of annotation tracks and the Diff Analysis tool, coupled with continuous data expansion, renders ChIP-Atlas 3.0 a robust resource for mining the landscape of transcriptional regulatory mechanisms, thereby offering valuable perspectives, particularly for genetic disease research and drug discovery.

## Graphical Abstract

**Graphical Abstract:**
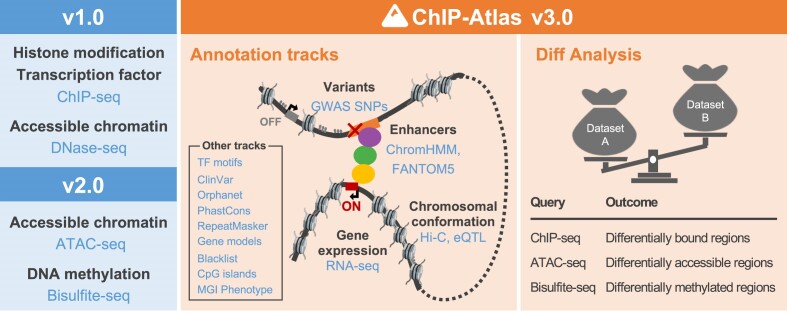

## Introduction

Multicellular organisms comprise different cell types with distinct phenotypes despite sharing a common genome in all somatic cells. This phenotypic diversity arises from cell type–specific gene expression and chromatin states orchestrated by genomic accessibility, DNA methylation status, histone modifications and transcription factors (TFs) binding to transcriptional regulatory elements. Numerous omics experiments have been performed using high-throughput sequencing to profile these characteristics, and the resulting raw sequencing data are available in public repositories.

The ChIP-Atlas project (https://chip-atlas.org/) was launched in 2015 to aid researchers without expertise in informatics in utilizing publicly available raw sequencing data. In ChIP-Atlas 1.0, the initial phase of our project (1), we performed sequence alignment and peak calling by downloading chromatin immunoprecipitation (ChIP-seq) and DNase I hypersensitive sites sequencing (DNase-seq) data for six model organisms, namely human, mouse, rat, fruit fly, nematode, and budding yeast, from public archives. Our web server offers pre-analyzed alignment and peak calling data, accompanied by a suite of data mining tools powered by this extensive dataset. This resource enables users to scrutinize TF bindings and histone modifications in single or multiple genomic regions using the Peak Browser or Enrichment Analysis tools, respectively. In the second phase of ChIP-Atlas (ChIP-Atlas 2.0) (2), we integrated tens of thousands of assays for transposase-accessible chromatin with sequencing (ATAC-seq) and whole-genome bisulfite sequencing (Bisulfite-seq) data. This expansion allows users to obtain panoramic views of chromatin accessibility and DNA methylation status in addition to protein–DNA interactions in genomic regions of their interest. In the ChIP-Atlas project, our focus up to now has been on meticulous curation, processing, and visualization of regulome and epigenome data, forming the molecular basis for understanding transcriptional regulatory mechanisms. Anticipating future research directions for unraveling the underlying mechanisms that govern cellular functions and responses, it is imperative to delve into the intricacies of chromatin architecture that mediate the regulome-initiated generation of transcriptional and phenotypic diversity within cells.

Here, we introduce ChIP-Atlas 3.0, the latest major update of ChIP-Atlas, which incorporates a variety of components that constitute part of the chromatin architecture along with cell- and tissue-specific gene expressions in a new ‘annotation tracks’ section in the Peak Browser tool. Furthermore, to address the need for comparative analysis between pre-processed regulome data, ‘Diff Analysis,’ a new online tool has also been developed and implemented on the ChIP-Atlas website. ChIP-Atlas 3.0 emerges as an attractive resource, offering a holistic understanding of transcriptional regulatory mechanisms.

## Materials and methods

### Data collection

The sample metadata described in the BioSample database of all experiments were downloaded from FTP sites of the National Center for Biotechnology Information (NCBI; ftp://ftp.ncbi.nlm.nih.gov/sra/reports/Metadata and ftp://ftp.ncbi.nlm.nih.gov/biosample) along with the monthly update of NCBI Sequence Read Archive (SRA). In the SRA, each experiment is assigned an ID prefixed with SRX, DRX, or ERX (hereafter referred to as SRX), which is also used in ChIP-Atlas for unified data management. ChIP-Atlas compiled the data from SRXs with the following criteria: LIBRARY_STRATEGY: ‘ChIP-Seq’, ‘ATAC-Seq’, ‘DNase-Hypersensitivity’, or ‘Bisulfite-Seq’; LIBRARY_SOURCE: ‘GENOMIC’; taxonomy_name: ‘*Homo sapiens*’, ‘*Mus musculus*’, ‘*Rattus norvegicus*’, ‘*Caenorhabditis elegans*’, ‘*Drosophila melanogaster*’ or ‘*Saccharomyces cerevisiae*’; and INSTRUMENT_MODEL: ‘Illumina’, ‘NextSeq’ or ‘HiSeq’. Sample metadata (ftp://ftp.ncbi.nlm.nih.gov/biosample/biosample_set.xml) were used to extract the attributes for ChIP antigens as well as cell or tissue types for each SRX. To structure the metadata, we sorted out notational distortions by submitters, which included manually annotating the names of both ChIP antigens and cell types, a task that was completed by well-trained curators who possess PhDs in molecular and developmental biology. Additional details can be found in our previous papers (1,2).

### Data aggregation to create annotation tracks

We incorporated more than a thousand genomic and epigenomic feature tracks sourced from external servers including UCSC goldenPath (3) encompassing six organisms. These features included chromosomal conformations (ENCODE Hi-C (4) and GTEx eQTL (5)), transcriptional regulatory elements (ChromHMM (6), FANTOM5 enhancers (7) and JASPAR TF motif (8)), genome-wide association studies (GWAS) identified SNPs (GWAS Catalog (9)), genetic variants (ClinVar (10)), gene–disease/phenotype associations (Orphanet (11) and MGI Phenotype (12)), evolutionary conserved regions (PhastCons (13)), repeated sequences (RepeatMasker (14)), RNA-seq-based transcriptomes (ENCODE (4), GTEx (5), FlyAtlas2 (15) and modENCODE (16)), gene models (Ensembl (17) and GENCODE (18)), and ENCODE Blacklist (19) and CpG Islands (https://genome-source.gi.ucsc.edu/gitlist/kent.git/tree/master/src/utils/cpgIslandExt/) (Table 1). The complete annotation track list and sources are listed in Supplementary Table S1. All the tracks compiled into an ‘annotation tracks’ section are conveniently accessible through the Peak Browser function on the ChIP-Atlas website.

**Table 1.** Available annotation tracks for each genome assembly

Genome	hg38	hg19	mm10	mm9	rn6	dm6	dm3	ce11	ce10	sacCer3
**ENCODE Hi-C** (4)	○	○	○	○						
**GTEx eQTL** (5)	○	○								
**ChromHMM** (6)	○	○	○	○						
**FANTOM5 enhancers** (7)	○	○								
**JASPAR TF motif** (8)	○	○	○	○		○	○	○	○	○
**GWAS Catalog** (9)	○	○								
**ClinVar** (10)	○	○								
**Orphanet** (11)	○	○								
**MGI Phenotype** (12)			○	○						
**PhastCons** (13)	○	○	○	○	○	○	○	○	○	○
**RepeatMasker** (14)	○	○	○	○	○	○	○	○	○	
**RNA-seq** (4,5,15,16)	○	○	○	○		○	○			
**Ensembl genes** (17)	○	○	○	○	○	○	○	○	○	○
**GENCODE genes** (18)	○	○	○	○						
**ENCODE Blacklist** (19)	○	○	○	○		○	○	○	○	
**CpG Islands** (*)	○	○	○	○	○	○	○	○	○	

*https://genome-source.gi.ucsc.edu/gitlist/kent.git/tree/master/src/utils/cpgIslandExt/.

### Implementation of the Diff Analysis tool

The Diff Analysis tool for detecting differentially bound regions (DBRs) from ChIP-seq or differentially accessible regions (DARs) from ATAC-seq and DNase-seq datasets is inspired by the R package ‘DiffBind’ (20). However, since DiffBind requires BAM files as input to perform comparative analysis, while BAM files are not available in the ChIP-Atlas server, we partially modified the algorithm for counting aligned reads within given ChIP-seq, ATAC-seq or DNase-seq peaks. In particular, upon user request, alignment data in bigWig format showing reads per million (RPM) were first converted to bedGraph format for each query SRX. RPMs were then converted to integer values concerning the total number of the mapped sequencing reads for the SRX. Next, the entire genome was fragmented based on the peak calling data from the query SRXs. We then aggregated the number of sequence reads aligning with each genome fragment and organized the result into an *m* × *n* matrix, with *m* representing the number of genome fragments and *n* representing the number of query SRXs. The matrix was then entered into the R package ‘edgeR’ (21), and the difference in read counts between the two sets of query SRXs was assessed for each genome fragment using the standard algorithm used for detecting differentially expressed genes in comparative transcriptome analysis. The outcomes were then further summarized and documented in BED format, which includes coordinates of the genome fragment in columns 1–3 and corresponding intergroup statistical values in columns 4 and beyond. The source code for the Diff Analysis tool to detect DBRs and DARs is available at https://github.com/inutano/chip-atlas/blob/master/script/diff_analysis_for_ChIP_ATAC_DNase.sh.

In the context of detecting differentially methylated regions (DMRs) from Bisulfite-seq datasets, it was also necessary to convert bigWig to bedGraph, which includes methylation rates for each query SRX. We used the DMR detector Metilene (22); in particular, ‘metilene_input.pl’ provided by metilene was used to aggregate methylation rates per genomic base for each query SRX, using the bedGraph of the query SRX generated in the prior step as input. The resulting TSV file was then used as the input into the main ‘metilene’ program, which returns DMRs along with statistics such as mean methylation differences and *Q*-values in a BED format. Default parameters were applied when executing both the ‘metilene_input.pl’ script and the ‘metilene’ command, in which the minimum mean methylation difference for calling DMRs was set to 0.1.

## Results

### Overview of the ChIP-Atlas 3.0 update

The ChIP-Atlas project aims to collect and analyze ChIP-seq, ATAC-seq, DNase-seq and whole-genome Bisulfite-seq data, originally archived in the NCBI SRA, along with associated sample metadata that have been manually curated by experts. Since the initial public release of ChIP-Atlas in 2015, the volume of data has steadily increased at a rate of approximately 3000 entries per month, in line with the monthly updates to the NCBI SRA (Supplementary Figure S1). The number of SRXs in ChIP-Atlas 3.0 exceeds 376 000 for six representative model organisms (ChIP-seq, *n* = 228 495; ATAC-seq, *n* = 84 615; DNase-seq, *n* = 6386; Bisulfite-seq, *n* = 56 668), which corresponds to 83.5% of the total number of SRXs using these sequencing technologies in NCBI SRA for all organisms. The unified processing pipeline identified over 11 billion genomic intervals (protein binding sites for ChIP-seq: *n* = 2 090 307 752; accessible genomic regions for ATAC-seq and DNase-seq: *n* = 1 297 948 075; hyper-, hypo and partially methylated regions for Bisulfite-seq: *n* = 7 885 144 497) (Table 2), which showed an approximately 30% increase compared with ChIP-Atlas 2.0 (2).

**Table 2.** Data statistics of ChIP-Atlas as of November 2023

Experiment type	Version	Number of experiments	Number of peaks	Number of cell types	Number of ChIP antigens
**ChIP-seq**	**1.0**	74 076	7.2 × 10^8^	1615	1979
	**2.0**	182 891	1.3 × 10^9^	2486	2477
	**3.0**	228 495	2.1 × 10^9^	3612	3945
**ATAC-seq**	**2.0**	66 104	3.6 × 10^8^	772	NA
	**3.0**	84 615	1.1 × 10^9^	1532	NA
**DNase-seq**	**1.0**	2050	1.0 × 10^8^	256	NA
	**2.0**	5346	1.3 × 10^8^	406	NA
	**3.0**	6386	1.8 × 10^8^	477	NA
**Bisulfite-seq**	**2.0**	51 074	3.8 × 10^9^	553	NA
	**3.0**	56 668	7.9 × 10^9^	731	NA
**All**	**1.0**	76 126	8.2 × 10^8^	1730	1979
	**2.0**	305 415	8.1 × 10^9^	2918	2477
	**3.0**	376 164	1.1 × 10^10^	4366	3945

Another notable highlight in ChIP-Atlas 3.0 is the addition of a new section in the Peak Browser tool termed ‘annotation tracks’, which consolidates features such as chromosomal conformation, transcriptional regulatory elements, and disease- or phenotype-associated genome variants. The annotation tracks can be visualized in the IGV genome browser (23) upon user request to the ChIP-Atlas web server, together with other Peak Browser track types. This is expected to help users understand the whole picture of molecular profiles, including the regulome, epigenome, and transcriptome, along with 3D chromatin architecture and DNA polymorphisms. In addition, we implemented the Diff Analysis tool, which allows users to identify statistically significant DBRs, DARs, and DMRs that have the potential to define transcriptional and phenotypic diversity in cells and tissues from two sets of ChIP-seq, ATAC-seq and DNase-seq, and Bisulfite-seq data, respectively. These functional enhancements in ChIP-Atlas 3.0 establish it as an increasingly comprehensive platform for a panoramic view of the cell fate determination process.

### Annotation tracks

Annotation tracks in ChIP-Atlas 3.0 can be graphically displayed with the IGV genome browser along with regulome data, including protein–genome interactions (ChIP-seq), chromatin accessibility (ATAC-seq and DNase-seq) and DNA methylation levels (Bisulfite-seq) within the queried genomic region of interest. To implement the annotation tracks, we collected and organized (a) chromosomal conformations such as ENCODE Hi-C and GTEx eQTL datasets (4,5), (b) transcriptional regulatory elements such as cell-specific promoters, enhancers, and heterochromatins from the ChromHMM and FANTOM5 projects, along with JASPAR TF-binding motifs (6–8), (c) disease– and phenotype–genome associations based on GWAS SNPs and ClinVar variants (9,10), (d) gene–disease/phenotype associations from Orphanet and MGI Phenotype (11,12), (e) conserved interspecies genetic sequences from PhastCons (13), (f) repeated sequences from RepeatMasker (14) and (g) RNA-seq-based transcriptome from the ENCODE, GTEx, FlyAtlas2 and modENCODE consortia (Table 1, Supplementary Table S1) (4,5,15,16). These features were then grouped into an ‘annotation tracks’ item arranged in the ‘Track type class’ option within the web interface of the ChIP-Atlas Peak Browser tool, which externally controls the IGV (23) preinstalled on the user's machine (tested on Mac, Windows, and Linux platforms). Users are recommended to run IGV on their own computer with at least 4 GB of RAM and an Internet speed of 100 Mbps.

**Figure 1.**
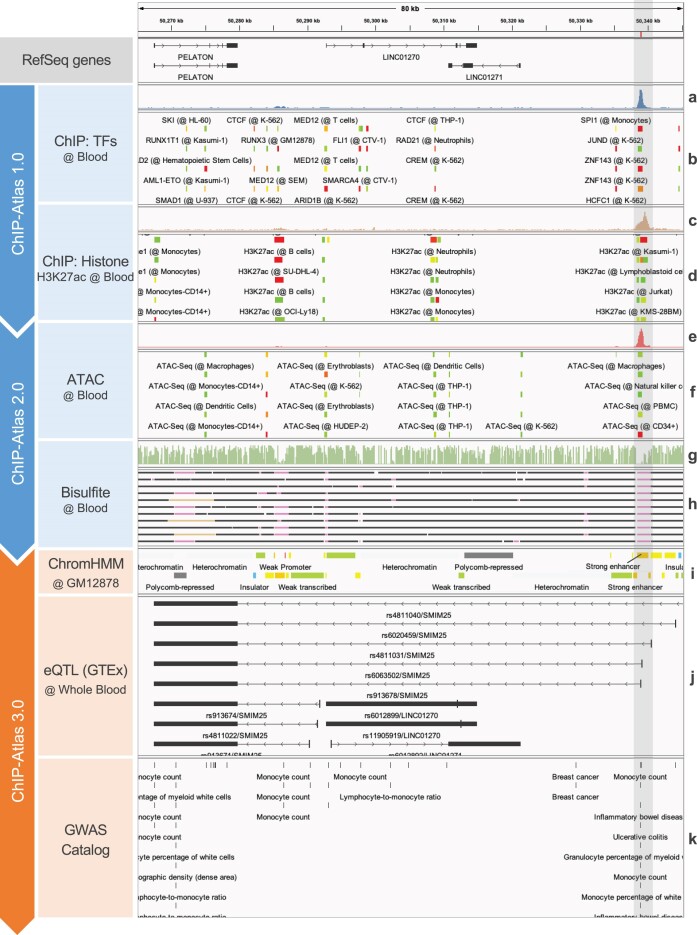
Example for browsing annotation tracks in ChIP-Atlas 3.0. Peak-call data for TF and histone ChIP-seq (ChIP-Atlas 1.0), ATAC-seq and Bisulfite-seq experiments (ChIP-Atlas 2.0), along with ChromHMM, eQTL and GWAS SNPs tracks (ChIP-Atlas 3.0) in human (hg38) blood around the *PELATON* locus are shown in the IGV genome browser. Panels a, c, e and f show single views of individual alignment data from ChIP-seq, ATAC-seq and Bisulfite-seq experiments (**a**, IKZF1 ChIP-seq in GM12878 [SRX2424550]; **c**, H3K27ac ChIP-seq in lymphoblastoid cell line [SRX4288387]; **e**, ATAC-seq in CD8^+^ T cells [SRX16731555]; **g**, methylation level in B cells [SRX9500396]) (see the tutorial PDF of Peak Browser [https://chip-atlas.dbcls.jp/data/manual/Peak_Browser/Peak_Browser.pdf] for more information on loading individual data for single views). Panels b, d, f and h show integrative views of TF (**b**) and histone (**d**) ChIP-seq peaks, ATAC-seq peaks (**f**) and hypo-, hyper- and partially methylated regions (**h**) in the cells categorized as ‘blood’ cell type class. The highlighted region (in gray) indicates an accessible chromatin and hypomethylated region. Bars in panels b, d and f represent the peak regions, the color of which indicates MACS2 scores (–10 × log_10_[*Q*-value]); i.e. if MACS2 scores are 50, 500 or over 1000, then the colors are blue, green or red, respectively. Panel h is shown in squished mode, and black, pink and beige bars indicate hyper-, hypo- and partially methylated regions, respectively. Colored bars in panel i indicate various chromatin states. Arrows from short bars to long bars in panel j indicate the effect of gene polymorphisms (short bars) on gene expression (long bars). Bars in panel k represent SNPs associated to diseases, phenotypes, measurements and drug responses. See Supplementary Figure S2A and B for details on the procedures for visualizing these tracks.

Here, we show an example of exploring annotation tracks together with the regulome data using Peak Browser. First, ChIP-seq (TFs and others) data of human (hg38) blood cells were specified in the query page of Peak Browser (Supplementary Figure S2A, B) by which the corresponding track was automatically streamed into IGV (Figure 1). Other experimental data, such as ChIP-seq (Histone: H3K27ac), ATAC-seq, and Bisulfite-seq, as well as annotation tracks (ChromHMM, eQTL, and GWAS SNPs) data of the same cell type class, were subsequently loaded using IGV in the same manner as the ChIP-seq (TFs and others) data. Individual alignment data from ChIP-seq, ATAC-seq, and Bisulfite-seq experiments were also shown in single views. The genomic region in the vicinity of *PELATON* (also known as *SMIM25*), a long noncoding gene reported as a biomarker and potentially involved in the pathogenesis of inflammatory bowel disease (IBD), is shown in Figure 1 (24,25). The eQTL data indicate that *PELATON* transcription is primarily influenced by genetic variations located approximately 70 kb downstream of the *PELATON* locus (highlighted). The co-localization of strong enhancers (ChromHMM) in conjunction with ATAC-seq, Bisulfite-seq, and H3K27ac peaks indicates that the region is protein accessible, hypo-methylated and with an ‘active’ histone modification in blood cells. In addition, the binding of TFs such as SPI1 and JUND, which contribute to hematopoietic differentiation and the release of pro-inflammatory signals, also suggests inflammatory activity within the genomic region. The presence of SNPs associated with IBD and monocyte count in this particular region is also noteworthy. Together with the above observations, we can make a reasonable inference that these SNPs may alter the chromatin landscape and disrupt normal TF binding in this region, thereby leading to a loss of control in *PELATON* transcription and resulting in the phenotypic manifestation of IBD. Examples of browsing other annotation tracks (Hi-C, RNA-seq, FANTOM5 enhancers, and JASPAR TF motifs) are shown in Supplementary Figure S3. IGV session is shown in Supplementary Materials S1 and S2.

### Diff Analysis

The Diff Analysis tool was implemented in ChIP-Atlas 3.0 as a brand-new online function offering both a graphical user interface (GUI) and application programming interface (API) to detect statistical differences between two groups of sequencing data upon query of SRX or GEO ID(s). Guidance on identifying IDs of interest to users is provided in the ‘Tips: IDs of experiments’ section of the tutorial at https://chip-atlas.dbcls.jp/data/manual/Diff_Analysis/Diff_Analysis.pdf. The calculation algorithm for DBRs (ChIP-seq) and DARs (ATAC-seq and DNase-seq) is inspired by the R package ‘DiffBind’ (20), while the detection of DMRs (Bisulfite-seq) is supported by the pre-existing ‘metilene’ tool (22) (see Materials and methods section for details). Calculation results are returned in BED format containing coordinates of genome regions with statistics such as *Q*-values.

**Figure 2.**
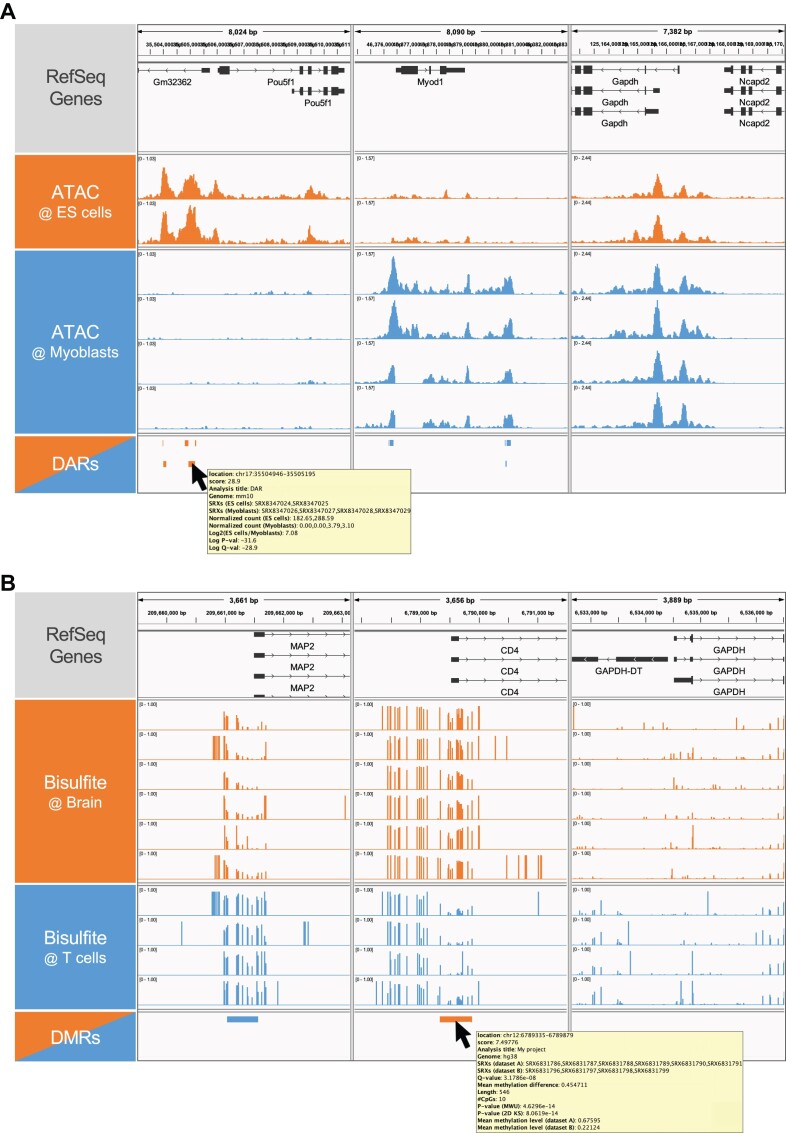
Example of visualizing the results of Diff Analysis tools. (A, B) Screenshot of IGV displaying the returned XML sessions containing alignment data of the query experiments along with the output DARs (A) and DMRs (B). The ‘DARs’ and ‘DMRs’ panels are shown in expanded mode. (**A**) Bars in the ‘DAR’ panel indicate ES cell- (orange) and myoblast-specific peaks (blue). (**B**) Bars in the ‘DMR’ panel indicate brain- (orange) and T cell-specific methylated regions (blue). DAR, differentially accessible region; DMR, differentially methylated region. Downloaded results for (A) and (B) are provided in Supplementary Materials S3 and S4, while details on the procedures for performing Diff Analysis are in Supplementary Figure S2C and D.

For example, after submitting the ATAC-seq experiment IDs under the experiment series SRA1075867 (26) in mouse embryonic stem cells (SRX8347024 and SRX8347025) and myoblasts (SRX8347026, SRX8347027, SRX8347028 and SRX8347029), the Diff Analysis tool returned a clickable HTML link to load the DAR data into IGV, along with a ZIP file consisting of a plain BED file (.bed) for further analysis, a BED9 + GFF3 format file (.igv.bed) for visualization using IGV, event logs (.log), and an IGV session XML file (.igv.xml) containing alignment data for queried SRXs and DARs (Supplementary Figure S2C, D). By loading the XML session file to IGV, DARs were clearly shown around the gene loci of *Pou5f1* (orange) and *Myod1* (blue), which are required for pluripotency and myogenic differentiation, respectively (Figure 2A, Supplementary Materials S3). Meanwhile, no DARs were detected around housekeeping *Gapdh* locus. In addition, we show an example of using Diff Analysis to detect DMRs between Bisulfite-seq data (SRA960814) (27) on human brain (SRX6831786, SRX6831787, SRX6831788, SRX6831789, SRX6831790 and SRX6831791) and T cells (SRX6831796, SRX6831797, SRX6831798 and SRX6831799). As a result, significant DMRs were detected around the vicinity of the transcription start sites for a subset of isoforms of *MAP2* (orange) and *CD4* (blue) encoding neuron-specific cytoskeletal proteins and T lymphocyte–specific surface glycoproteins, respectively (Figure 2B, Supplementary Materials S4). Meanwhile, no DMRs were detected around housekeeping *GAPDH* locus. These cases mentioned above suggest that Diff Analysis is capable of identifying key differences in the epigenomic landscape that define transcriptional and phenotypic diversity in cells.

In addition to the pre-analyzed data, Diff Analysis can accommodate user-generated data stored on custom web servers that are publicly accessible. The procedure to perform analysis on their data is outlined in the ‘Diff Analysis’ section of the documentation and the tutorial PDF (https://chip-atlas.dbcls.jp/data/manual/Diff_Analysis/Diff_Analysis.pdf). In brief, users must make a query by inputting the URLs of raw read coverage (bigWig format) and peak-call (BED format) data and total number of mapped reads for ChIP-, ATAC-, and DNase-seq, and bigWig data of methylation rate (between 0 and 1) for Bisulfite-seq.

## Discussion

In addition to an extensive increase in the number of SRXs, this paper presents a significant ChIP-Atlas update, specifically centering on offering annotation tracks and a Diff Analysis tool for users to attain a comprehensive understanding of the chromatin architecture associated with transcriptional regulatory mechanisms that have the potential to impact cell fate determination.

As with ChIP-Atlas, a number of other similar web services have also been made available, of which Cistrome DB (https://db3.cistrome.org/browser/) (28), ReMap (https://remap2022.univ-amu.fr/) (29), and GTRD (https://gtrd.biouml.org/) (30) offer an array of pre-analyzed ChIP-seq and chromatin accessibility data sets, numbering in the tens of thousands, while MethBank (https://ngdc.cncb.ac.cn/methbank/) (31) is known for assembling data from hundreds of methylome analysis projects (Table 3). The amount of experimental data in ChIP-Atlas surpasses that of all other services. Quality control filtering is not performed on data in the ChIP-Atlas project. Instead, expert-curated sample metadata is furnished for each SRX, enabling users to independently assess the robustness of their selected SRX if necessary. ChIP-Atlas only covers ChIP-seq, ATAC-seq, DNase-seq and whole-genome bisulfite-seq data sets in six organisms, while certain additional experimental methods, such as ChIP-exo, MNase-seq, and FAIRE-seq, and organisms such as plants, are still to be supported. Differences between SRXs can be detected using ChIP-Atlas, which provides both a user-friendly GUI and programmable API for batch processing (refer to the ChIP-Atlas documentation). On the contrary, MethBank only offers a tool for analyzing DMRs that need to be run locally in a command-line interface. The ChIP-Atlas website is free and open to all users without requiring login credentials. All data and analysis tools provided by ChIP-Atlas are available for unrestricted use in non-commercial and commercial contexts, given that proper citation is provided.

**Table 3.** Comparison of ChIP-Atlas with other similar services

	ChIP-Atlas		Cistrome DB		ReMap		GTRD		MethBank	
Year	2022	2024	2022	2024	2022	2024	2022	2024	2022	2024
**Data source**	NCBI SRA		GEO, ENCODE and Roadmap		GEO, ENCODE and ENA		GEO, SRA, ENCODE and modENCODE		NCBI SRA and Genome Sequence Archive	
**Experiments**	ChIP-seq, ATAC-seq, DNase-seq and Bisulfite-seq		ChIP-seq, ATAC-seq and DNase-seq		ChIP-seq, ChIP-exo and DAP-seq		ChIP-seq, ChIP-exo, ChIP-nexus, MNase-seq, DNase-seq, FAIRE-seq, ATAC-seq, and RNA-seq	**+ Bisulfite-seq**	Bisulfite-seq	
**Data filtering for quality control before alignment**	No		Yes		Yes		No		Yes	
**Number of experiments**	305 415	**376 164**	56 442	**∼90 000**	19 983		36 540	**106 397**	673	**1449**
**Organism**	*Hs*, *Mm*, *Rn*, *Dm*, *Ce* and *Sc*		*Hs* and *Mm*		*Hs*, *Mm*, *Dm*, and *At*		*Hs*, *Mm*, *At*, *Ce*, *Dr*, *Dm*, *Rn*, *Sc* and *Sp*	***+ Gga***	*Hs*, *Dr*, *Mm*, *Os*, *Gm*, *Me*, *Pv* and *Sl*	**+ *Am*, *At*,*Bt*, *Bn*, *Cl*, *Gga*, *Ggo*, *Mf*, *Mmul*, *Oa*, *Ptro*, *Ptri*, *Rn, Ssa*, *Ssc*, *Xl* and *Zm***
**Integrative analysis tools**	Search tool for target genes and colocalizing factors of given TF, and enrichment analysis tool for given genes and genomic coordinates	**+ Annotation tracks for various chromosomal architectures and Diff Analysis tool for detecting differential peaks or differentially methylated regions from query experiments**	Search tool for target genes of single experiment, TFs binding to single given genomic locus or query gene, and gene ontology enrichment analysis tool (Cistrome-GO)	**Addition of a toolkit, including search tool for TF target genes based on a single experiment or genomic locus and for factors with a significant binding overlap with query peak set**	Enrichment analysis tool for given genomic coordinates relative to random background		Search tool for target genes of given TF and binding site prediction tool based of TF motifs		Predictor of DNA methylation age of human blood and enrichment analysis tool for the identification of differentially methylated promoters	**+ CLI-based DMR detector**
**Requirement of login**	No		Yes (for batch download)		No		Requires a license for commercial purpose		No	

*Hm, Homo sapiens; Mm, Mus musculus; Rn, Rattus norvegicus; Dm, Drosophila melanogaster; Ce, Caenorhabditis elegans; Sc, Saccharomyces cerevisiae; At, Arabidopsis thaliana; Dr, Danio rerio; Sp, Schizosaccharomyces pombe; Gga, Gallus gallus; Os, Oryza sativa; Gm, Glycine max; Me, Manihot esculenta; Pv, Phaseolus vulgaris; Sl, Solanum lycopersicum; Am, Ailuropoda melanoleuca; Bt, Bos taurus; Bn, Brassica napus; Cl, Canis lupus familiaris; Ggo, Gorilla gorilla; Mf, Macaca fascicularis; Mmul, Macaca mulatta; Oa, Ovis aries; Ptro, Pan troglodytes; Ptri, Populus trichocarpa; Ssa, Salmo salar; Ssc, Sus scrofa; Xl, Xenopus laevis; Zm, Zea mays*; CLI, command line interface.

Since its public release, ChIP-Atlas has been utilized in diverse research areas, including genetics, etiology, developmental biology, and drug discovery and cited in over 700 publications (https://chip-atlas.org/publications for full publication list). The updated ChIP-Atlas 3.0 is expected to provide valuable insights into, for instance, genetic disease research by incorporating chromosome architecture data, thus examining and providing a comprehensive view of transcriptional regulatory mechanisms. Although numerous susceptibility SNPs for inherited diseases have been identified through GWAS, how these SNPs alter gene expression and contribute to disease development is not completely understood, as most of them are found in non-coding regions. To overcome this challenge, we previously conducted an enrichment analysis using large-scale ChIP-seq experimental data in ChIP-Atlas and were able to successfully identify TFs that exhibit enriched binding to SNPs associated with atrial fibrillation (32). By further utilizing the Hi-C and eQTL tracks from ChIP-Atlas 3.0, a systematic elucidation of a cascade of events should be possible, in which the presence of disease-associated SNPs induces anomalous TF binding, consequently resulting in gene expression abnormalities within certain chromosomal conformations. Apart from genetic disease research, we also analyzed TF binding that was enriched in DARs induced by chemical exposure to identify pivotal TFs involved in chemical action modes (33). Because the detection of DARs in the approach proposed in this study requires the use of external command-line-interface tools, the methodology may be challenging to implement for those without expertise in genome informatics. Nevertheless, after implementing the Diff Analysis tool in ChIP-Atlas 3.0, the entire pipeline becomes readily accessible on the ChIP-Atlas website, thereby significantly contributing to drug discovery research.

The amount of experimental data available on ChIP-Atlas is steadily increasing with consistent monthly updates and expert curation, and the annotation data are to be updated periodically. Our future plan is to further expand ChIP-Atlas with additional experiment types, like CUT&Tag (34) and ChIL-seq (35), and organisms, including fish, plants and non-human primates. Furthermore, to address spatio-temporal gene expression in cells of multicellular organisms, the integration of data from spatial epigenetics technologies is also under active consideration.

## Supplementary Material

gkae358_Supplemental_Files

## Data Availability

ChIP-Atlas (https://chip-atlas.org/) is an open-source web service. All data is freely available for both non-commercial and commercial purposes. See the documentation (https://github.com/inutano/chip-atlas/wiki/ and https://doi.org/10.5281/zenodo.11044059) for details.
