# Supplementary material for: ChIP-Atlas 3.0: a data-mining suite to explore chromosome architecture together with large-scale regulome data
Source: Nucleic Acids Res. 2024 May 16;52(W1):W45–53. doi: 10.1093/nar/gkae358 (PMC11223792; doi:10.1093/nar/gkae358)
Supplement: gkae358_Supplemental_Files [file gkae358_supplemental_files.zip › SuppFigure.pdf]

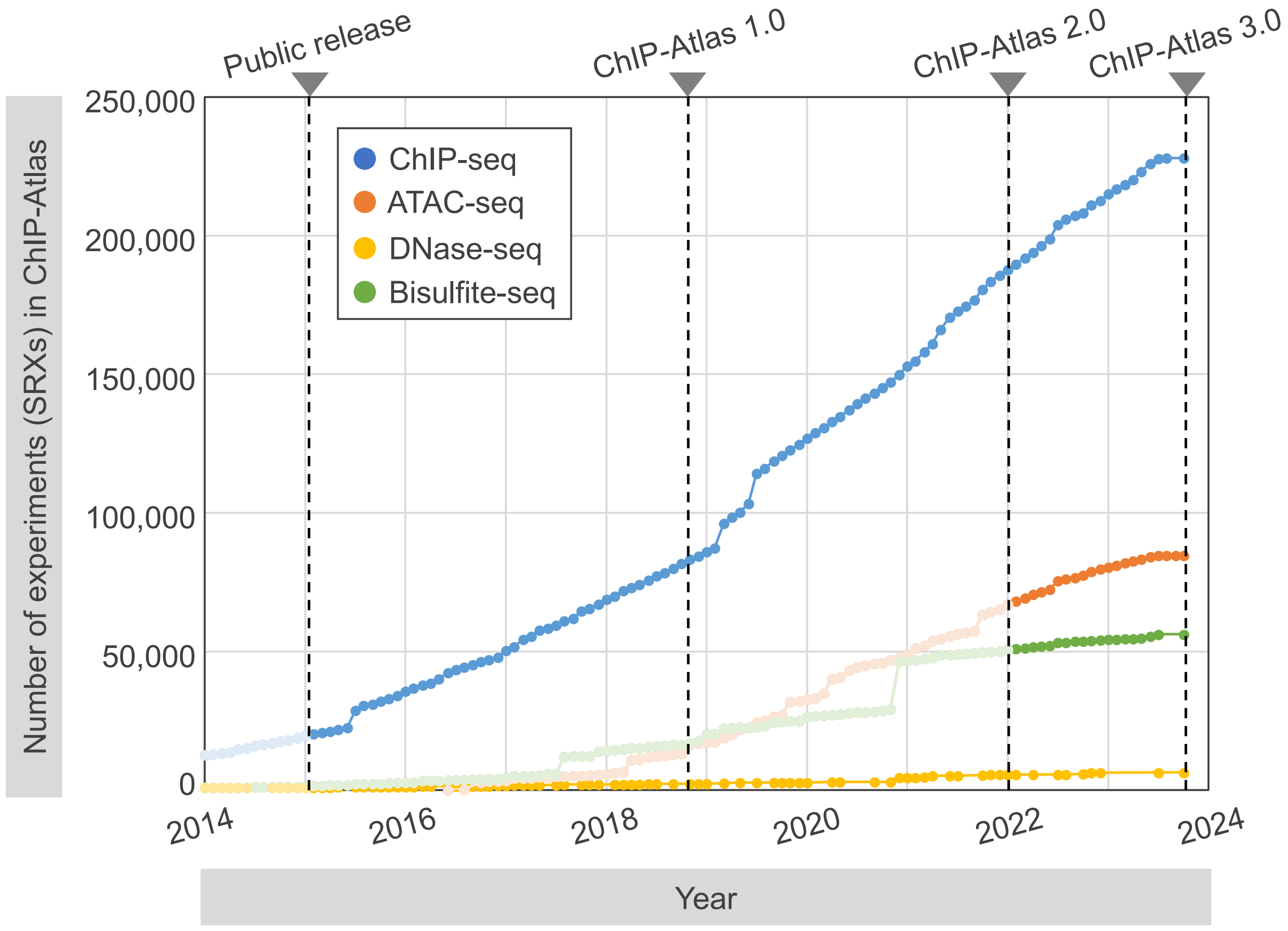

Supplementary Figure S1

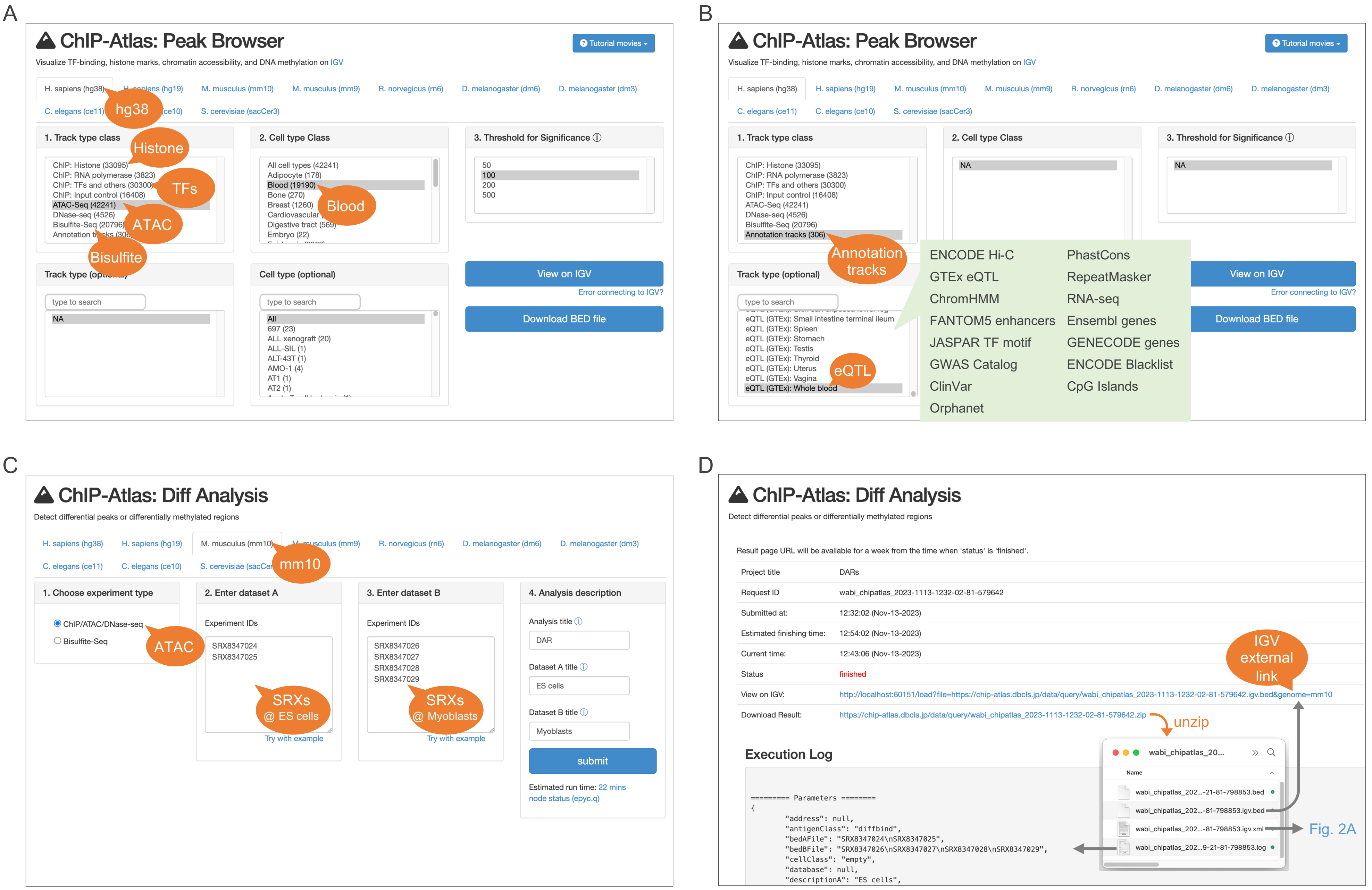

Supplementary Figure S2

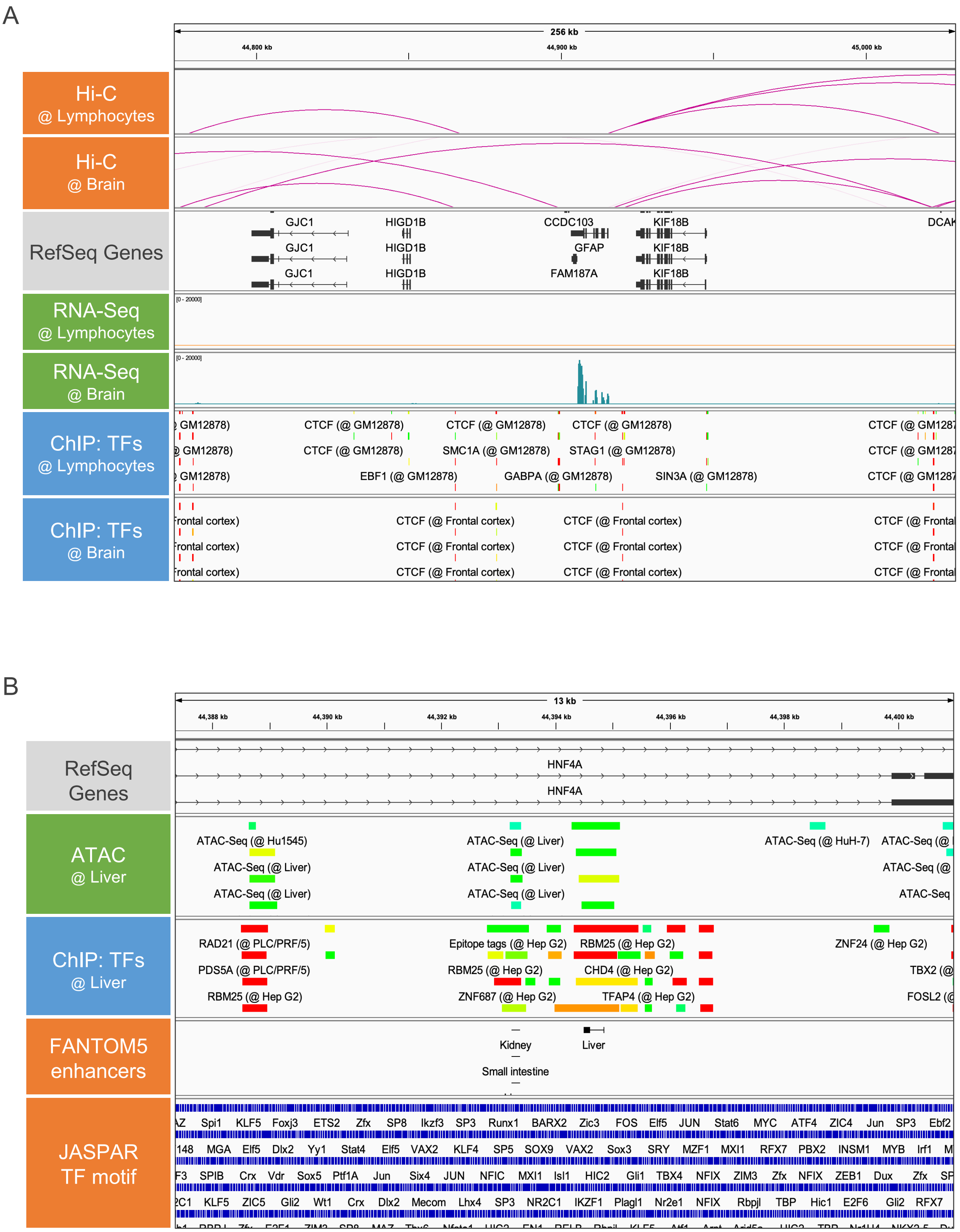

Supplementary Figure S3
