# Supplementary material for: ChIP-Atlas 3.0: a data-mining suite to explore chromosome architecture together with large-scale regulome data
Source: Nucleic Acids Res. 2024 May 16;52(W1):W45–53. doi: 10.1093/nar/gkae358 (PMC11223792; doi:10.1093/nar/gkae358)
Supplement: gkae358_Supplemental_Files [file gkae358_supplemental_files.zip › SuppLegend.docx]

## SUPPLEMENTARY DATA

Supplementary Data are available at NAR online.

**Supplementary Figure S1**. Cumulative number of experiments in ChIP-Atlas. Colors of the dots indicate different experiment types. ChIP-seq and DNase-seq data published before the public release of ChIP-Atlas in 2015 and ATAC-seq and Bisulfite-seq data published before the ChIP-Atlas 2.0 update in 2022 are shown in lighter tones.

**Supplementary Figure S2**. **(A, B)** Parameters for a query to show TF binding, histone modification, chromatin accessibility, methylation status, and annotation tracks around human *PELATON* locus in blood samples using the Peak Browser tool. **(C)** Parameters when users perform a query to detect DARs from two sets of ATAC-seq experiments. **(D)** Result page of Diff Analysis.

**Supplementary Figure S3**. Additional examples for browsing annotation tracks in ChIP-Atlas 3.0 using IGV. **(A)** Hi-C (Hi-C (ENCODE): GM12878_1; Hi-C (ENCODE): Dorsolateral prefrontal cortex) and RNA-seq (RNA-seq (ENCODE): GM12878, rep 1; RNA-seq (GTEx): Brain, cortex) tracks in human lymphocytes and brains around *GFAP* locus, a marker of reactive astrocytes, along with TF ChIP-seq peaks in the same tissues (ChIP: TFs and others, Blood, GM12878; ChIP: TFs and others, Neural, Frontal cortex). **(B)** ATAC-seq (ATAC-seq, Liver) and TF ChIP-seq (ChIP: TFs and others, Liver) peaks in human liver around *HNF4A* locus along with FANTOM5 expressed enhancers and JASPAR TF motifs. Bars in the “ChIP: TFs” and “ATAC” panels in (A) and (B) present the peak regions, the color of which indicates MACS2 scores (–10 × log_10_[*Q*-value]); i.e., blue, green, or red colors correspond to MACS2 scores of 50, 500, or over 1000, respectively. IGV session files for (A) and (B) are provided as Supplementary Materials S1 and S2.

**Supplementary Table S1**. Detailed list of annotation tracks. The strings “_CHROMOSOME_” in the RepeatMasker source should be replaced by chromosome numbers, e.g., “chr2L” for dm3 and “chr3” for mm9.

**Supplementary Materials S1.** IGV session file (XML) for Supplementary Figure S3A.

**Supplementary Materials S2.** IGV session file (XML) for Supplementary Figure S3B.

**Supplementary Materials S3.** Downloaded ZIP file for Figure 2A.

**Supplementary Materials S4.** Downloaded ZIP file for Figure 2B.
